# Oligodendroglia are emerging players in several forms of learning and memory

**DOI:** 10.1038/s42003-022-04116-y

**Published:** 2022-10-29

**Authors:** Maxime Munyeshyaka, R. Douglas Fields

**Affiliations:** 1 National Institute of Mental Health (NIMH), National Institutes of Health (NIH), Bethesda, MD 20892 USA; 2 Section on Nervous System Development and Plasticity, The Eunice Kennedy Shriver National Institute of Child Health and Human Development (NICHD), National Institutes of Health (NIH), Bethesda, MD 20892 USA

**Keywords:** Learning and memory, Oligodendrocyte

## Abstract

Synaptic plasticity is the fundamental cellular mechanism of learning and memory, but recent research reveals that myelin-forming glia, oligodendrocytes (OL), are also involved. They contribute in ways that synaptic plasticity cannot, and the findings have not been integrated into the established conceptual framework used in the field of learning and memory. OLs and their progenitors are involved in long-term memory, memory consolidation, working memory, and recall in associative learning. They also contribute to short-term memory and non-associative learning by affecting synaptic transmission, intrinsic excitability of axons, and neural oscillations. Oligodendroglial involvement expands the field beyond synaptic plasticity to system-wide network function, where precise spike time arrival and neural oscillations are critical in information processing, storage, and retrieval.

## Abstract (web summary)

A Perspective highlights current evidence that supports oligodendrocytes and their progenitors’ involvement in cognition and proposes that our understanding of learning and memory can be expanded beyond the classic view of synaptic plasticity to a system-wide network function.

## Introduction

Recently the involvement of a type of non-neuronal cell, oligodendroglia^1^, in several forms of learning has become evident from both human and animal studies. These glial cells form the myelin sheaths on axons in the central nervous system, a process that is traditionally considered outside the context of synaptic plasticity in learning. Although controversial at first, it is now well established that oligodendroglia can detect neural impulse activity to modify the formation of myelin according to neural circuit function and sensory experience. Moreover, the structure of myelin can change during learning to alter the speed of impulse transmission. This introduces a new concept into the field of learning and memory, in which the speed and synchrony of action potential propagation rather than the amplitude of the postsynaptic potential, is an important electrophysiological aspect of information encoding, storage, and retrieval. In addition, immature oligodendrocyte progenitor cells (OPCs), which represent 5%-8% of total cells in the adult brain^2^, are widely expressed throughout both grey and white matter regions, and they can receive synaptic inputs from excitatory^3^ or inhibitory neurons^4^. Together progenitors and mature oligodendrocytes contribute to learning and memory by influencing synaptic transmission, axon excitability, and by myelination to modify the speed of impulse conduction.

### Encoding, storage, and retrieval of information: spike time arrival and oscillations

**Fig. 1:**
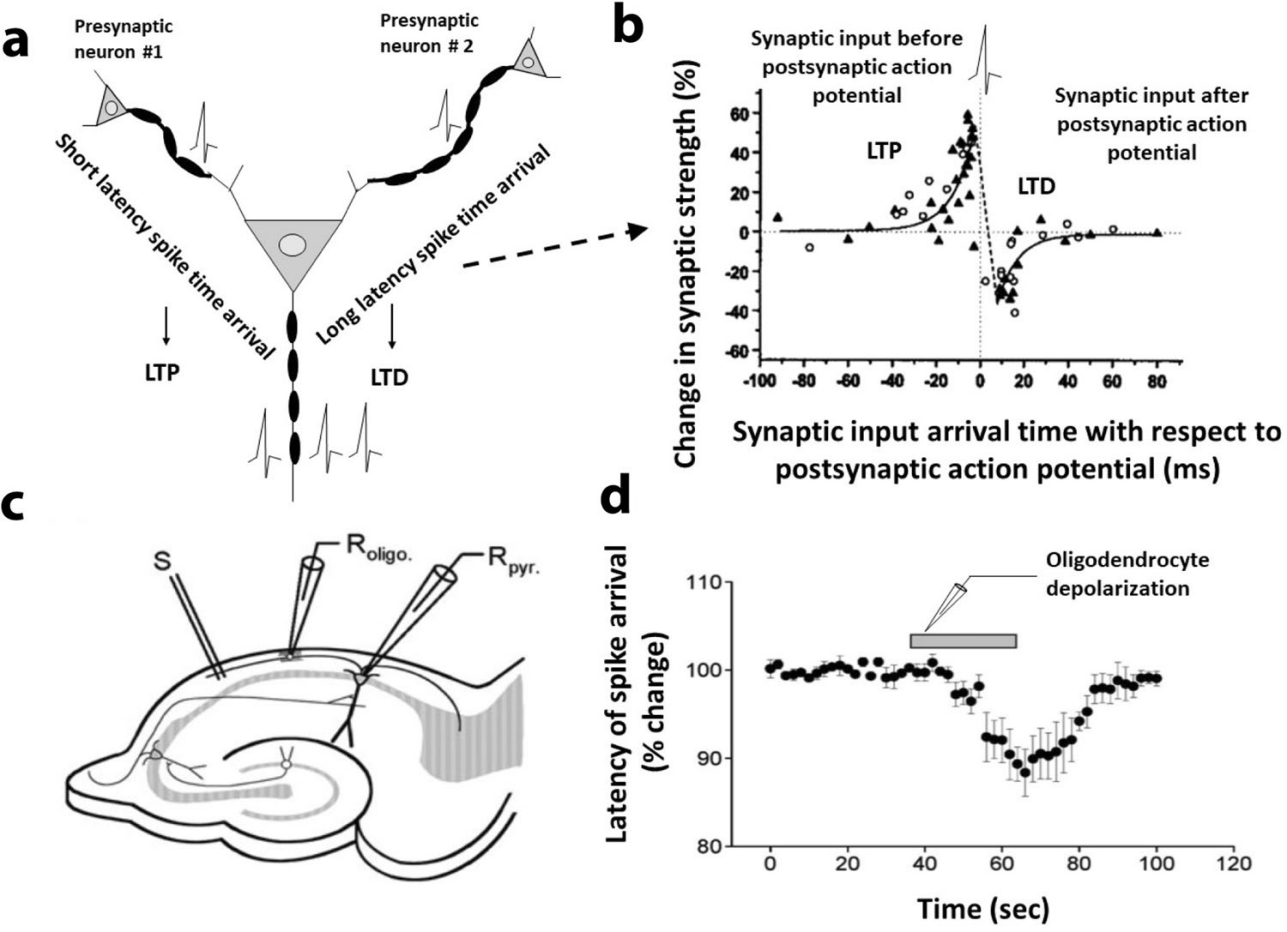
Oligodendrocytes in spike-timing-dependent plasticity. By altering conduction velocity in afferents converging onto a postsynaptic neuron, myelin plasticity can induce spike-timing-dependent synaptic plasticity by determining the synchrony of spike time arrival with respect to postsynaptic action potential firing. **a** Effect of action potential latency in spike time arrival on synaptic plasticity. Arrival of synaptic input coincident with or prior to postsynaptic neuron firing, due to rapid conduction velocity or shorter axon path length, will result in long-term potentiation (LTP). Longer latency in spike time arrival, due to slower conduction velocity or longer axon path length, will lead to long-term depression (LTD). **b** Critical window for synaptic LTP and LTD in *Xenopus* tectal neurons. The percent change in the EPSC of synaptic inputs after repetitive stimulation (at 1 Hz for 100 s) plotted against the time of action potential input. Open circles represent repetitive spiking induced by injection of depolarizing currents. Impulses arriving within 20 ms before postsynaptic neuron firing become strengthened (i.e., LTP) while impulses arriving 20 ms after postsynaptic neuron firing are weakened (i.e, LTD). Adapted from Zhang et al.^94^, with permission. **c**, **d** Oligodendrocytes modulate conduction velocity. **c** Dual, whole cell recoding in hippocampal CA1 pyramidal neurons and oligodendrocytes. **d** Time-course of the latency of action potential arrival after depolarization of an oligodendrocyte forming myelin on that axon. The gray bar indicates time when oligodendrocytes were depolarized. Depolarization of oligodendrocytes increases action potential conduction velocity, and theta burst stimulation of axons depolarizes oligodendrocytes through glutamate receptors and potassium channels. Error bars = SEM. Adapted from Yamazaki et al., (2007)^32^ with permission.

Learning and memory involve encoding, storage, and retrieval of information in neural circuits, and oligodendroglia contribute to each process. From the perspective of synaptic plasticity, information is encoded according to the Hebbian learning rule, in which the synchronous arrival of impulses from axons converging onto a common target initiates a mechanism that strengthens the coincidently active synaptic connection^5^. Conversely, synapses that are activated out of synchrony with firing of the postsynaptic neuron are weakened^6^. This experience-dependent plasticity forms functional assemblies of neural circuits that encode diverse aspects of experience into a coherent schema. But what drives this process of synaptic modification is action potential firing. The time window over which synaptic inputs must be activated in synchrony with firing of the postsynaptic neuron to strengthen or weaken a synapse is narrow^7^. Impulses arriving at dendrites simultaneously or about 20 ms before firing of the postsynaptic action potential will be strengthened, but synapses will be weakened if they are activated within about 20 ms after the postsynaptic neuron fires (Fig. 1). This millisecond precision of spike time arrival is very brief with respect to the long latencies of transmission through CNS networks, which typically requires tens or hundreds of milliseconds.

#### Spike time arrival

Since the time of arrival of an action potential depends on the axon path length and the speed of action potential propagation, oligodendrocytes, which greatly increase conduction velocity by forming myelin, have a fundamental role in encoding information via the Hebbian mechanism. If the conduction velocity in an axon cannot achieve synchronous spike time arrival, synapses from that axon will be weakened and eventually lost. It follows that if conduction velocity is altered by myelination or other influences of oligodendrocytes on axons, then oligodendroglia would initiate plasticity of synaptic transmission that determines the encoding of information via Hebbian rules. A recent study used transgenic mice with extra copies of proteolipid protein 1 gene *(PLP-tg)*, which results in thinner myelin, showed that action potentials become less synchronized in motor cortex during motor learning compared to wild-type animals^8^. Similarly, dynamic changes in myelin thickness and node of Ranvier structure in the adult optic nerve that slow conduction velocity increase the latency of spike time arrival in the visual cortex and reduce visual acuity^9^. Although myelin has many functions that could influence learning, including maintaining axon survival^10^, optogenetic stimulation of thalamocortical axons paired with learned forelimb movements in the studies by Kato et al.^8^, show that myelin contributes to motor learning by promoting synchrony of spike time arrival.

#### Neural oscillations

**Fig. 2:**
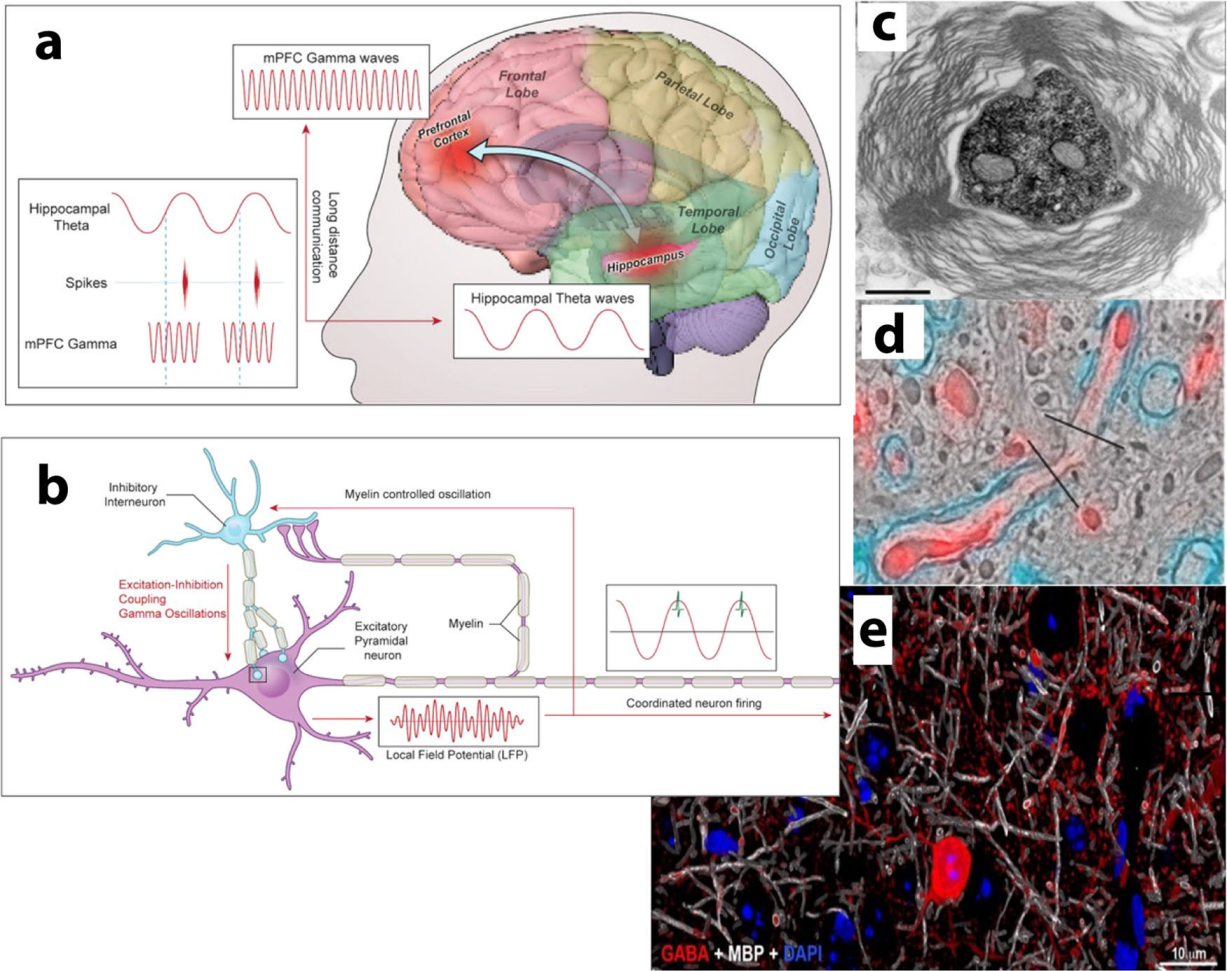
Myelin plasticity contributes to learning and memory by altering the frequency, phase and amplitude coupling of neural oscillations. **a** Conduction latency will have a profound influence on phase and frequency coupling of neural oscillations between distant brain regions that are critical for memory induction and recall. **b** The frequency of oscillations in local circuits, such as gamma oscillations in cerebral cortex, will be affected by conduction velocity in axons between inhibitor and excitatory neurons. Excitatory-inhibitory coupling between inhibitory interneurons and excitatory pyramidal neurons generate gamma oscillations. The frequency of oscillation is affected by the strength and latency of synaptic transmission, which is influenced by myelination of axons on inhibitory and excitatory neurons. Action potential firing (green) is entrained by the phase and frequency of subthreshold neural oscillations and local field potentials. **c** Myelinated projecting axons from inhibitory neurons in CA1 of the hippocampus to the subiculum. Myelin sheath thickness and axon diameter are larger in axons from hippocampal inhibitory neurons that project over long distances than CA1 axons from excitatory neurons to provide more rapid conduction velocity to sustain neural oscillations at appropriate phase and frequency between hippocampus and cortical regions participating in memory formation and recall. The apparent loose compaction of myelin is an artifact of fixation necessary for immunocytochemistry. Reprinted from Jinno et al., (2007)^95^ with permission (copyright 2007 Society for Neuroscience). **d** Scanning electron micrograph of a node of Ranvier on an axon of an interneuron in the cerebral cortex, immunolabeled with myelin basic protein (cyan) and GABA (red). **e** A large fraction of inhibitory interneurons are myelinated in cerebral cortex. GABA (red), myelin basic protein (white), nuclei stained with DAPI (blue). **d**, **e** reprinted from Micheva et al., (2016)^18^ with permission.

In addition to the importance of spike time arrival, neural oscillations influence the integration of synaptic potentials. The firing of action potentials is promoted or inhibited by subthreshold transmembrane voltage oscillations that move the neuron closer or farther from the threshold for action potential activation. In this way, neuronal oscillations coordinate synaptic integration and action potential firing in populations of neurons by amplitude, frequency, and phase coupling. Induction of LTP (long-term potentiation), considered a cellular substrate of short-term and long-term memory, is facilitated in the hippocampus by theta oscillations at ~5 Hz^11^. A brief stimulus given at the peaks of theta oscillations induces homosynaptic LTP, but the same stimulus delivered at troughs in theta oscillations induces homosynaptic LTD (long-term depression) of previously potentiated synapses. Thus, changes in conduction velocity will affect spike time arrival with respect to neural oscillations and thus synaptic plasticity. Only about 75 ms difference in the timing of action potential arrival with respect to the phase of theta oscillations will determine whether a synapse is maximally strengthened or maximally weakened. At gamma frequencies (30–90 Hz), much higher precision is required. The period of gamma oscillations also coincides with the temporal window of spike time-dependent synaptic plasticity (Fig. 2).

Cross frequency interactions between gamma oscillations of different frequencies and with slower rhythms such as theta oscillations orchestrate information processing and transmission by coupling activity in neurons transiently among distant brain regions that encode different aspects of experiences that are critical for learning and memory (Fig. 2). For example, the lateral and medial entorhinal cortex receive inputs from two distinct cortical sources providing what and where information to the hippocampus during learning. Optogenetic perturbation of these gamma oscillations results in selective impairments of spatial and object-related learning^12^. Similarly, neural oscillations couple populations of neurons during learning to integrate space and time (for review see ref. ^13^), combine sensory modalities, provide situational context, and contribute emotional value to encode experiences to induce learning^14^.

Several mechanisms of gamma oscillations are known (for review see ref. ^15^), but until recently, the importance of myelin was not appreciated. Myelination contributes to optimization of oscillations in local circuits and in coupling oscillations between distant brain regions (Fig. 2). Reciprocal connections between populations of excitatory and inhibitory neurons in neocortex and hippocampus produce oscillations in the gamma frequency range (Fig. 2b), and the oscillation frequency depends on the excitatory/inhibitory balance^16^. Mutual inhibition among inhibitory interneurons also generates gamma oscillations^17^, and as with coupling between excitatory and inhibitory neurons, the frequency of oscillation depends on the delay and rise time of the synaptic potentials. Axons of excitatory projecting neurons are myelinated to promote long distance saltatory conduction, but surprisingly, half of the total myelin in layers 2/3 and a quarter in layer 4 of the cortex is on inhibitory interneurons^18^. Myelin on inhibitory fast-spiking interneurons is critical for feedforward inhibition of cortical circuits^19^, which would alter local cortical circuit oscillations. In addition to effects on local circuits, myelination of axons from inhibitory neurons projecting long distances also coordinates neural oscillations and action potential firing between distant brain regions, for example, between the CA1 hippocampal region and subiculum/entorhinal cortex (Fig. 2c)^15^. Therefore, myelination and changes in myelin will affect functional coupling of neural oscillations between distant brain regions (Fig. 2a) and also within local circuits (Fig. 2b) that are critical for learning and memory. Changes in conduction velocity resulting from alterations in myelination of excitatory and inhibitory axons, and other effects of oligodendroglia on axon conduction velocity (discussed below), will thereby influence memory induction and recall.

This is supported by mathematical modeling of the effect of conduction delays between coupled neural oscillators^20^. The modeling indicates that there must be activity-dependent mechanisms to alter conduction velocity to prevent destructive interference and to facilitate coordinating oscillatory activity among populations of neurons that are separated by conduction delays. This is consistent with mathematical modeling indicating that activity-dependent myelin plasticity can promote phase synchrony of neural oscillations^21^. Experimental verification that conduction latencies have a critical influence on the frequency, phase, and coupling of oscillatory neural activity in learning has been provided recently in studies on the effects of myelination on spatial learning and fear conditioning in mice^22^.

Myelination also affects the pattern of structural connectivity in neural circuits. A recent study reports that a loss of compact myelin induced by cuprizone treatment reduces the number of presynaptic terminals from parvalbumin-positive interneurons and alters gamma and theta oscillations^23^. Consistent with this, a specific loss of parvalbumin-positive neurons is seen in the cortex and hippocampus in a cuprizone mouse model of demyelination and in multiple sclerosis patients^24,25^, which is accompanied by alterations in delta and theta rhythms^26,27^. Thus, oligodendroglia can affect gamma and theta oscillations in local circuits through myelination and by altering the number of synapses. The mechanisms by which alterations in myelin change synaptic connectivity are being investigated, but they likely include trophic factors and influences on axonal branching, synaptogenesis, and synapse elimination. Axons in grey matter frequently have intermittent myelination with long stretches of unmyelinated axons interspersed with myelinated segments^28^. In these local circuits, the intermittent myelination of axons may reflect oligodendrocyte sculpting of neural network connectivity by suppressing axon branching and synapse formation to sustain optimal neural oscillations and local circuit function to process information and encode experience.

#### Spike rate and temporal coding

**Fig. 3:**
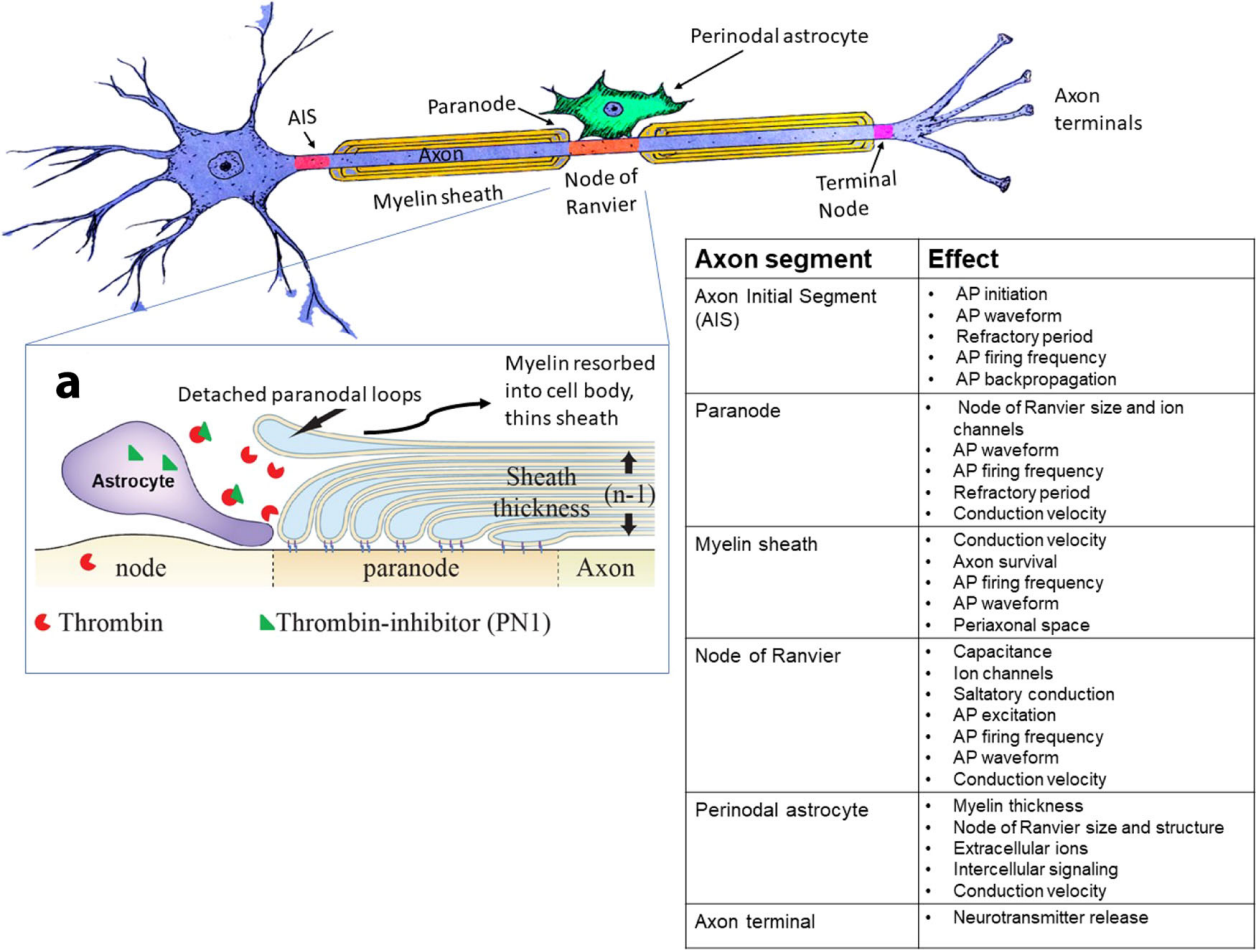
Cellular mechanism of oligodendroglial involvement in memory storage. Encoding and storage of information in learning are influenced by oligodendroglial involvement in many aspects of action potential firing and propagation. In addition to altering conduction velocity through activity-dependent myelination and plasticity of the myelin sheath, activity-dependent effects on the axon initial segment, nodes of Ranvier, axon terminals, and interactions with perinodal astrocytes will determine action potential activation, firing frequency, refractory period, and other aspects of neuronal excitation that will participate in information encoding and storage in non-associative and associative learning. **a** Shows widening of the nodal gap in mature myelin to reduce conduction velocity by lifting of the paranodal loops of myelin. This mechanism of widening the nodal gap and thinning the myelin sheath is under control of the perinodal astrocytes by releasing protease inhibitors (green) to inhibit thrombin (red) cleavage of the cell adhesion molecules attaching myelin to the axon. Modified from Fields and Dutta (2019)^96^, with permission.

Oligodendrocytes, by forming myelin and establishing the nodes of Ranvier, also have a central role in information encoding and storage through alterations determining neuronal spike coding (Fig. 3). Spike rate coding of information (action potential firing frequency) and temporal coding (spike arrival times) are limited by action potential activation threshold, firing rate, refractory periods, and conduction velocity.

The axon initial segment (AIS) is the first node of Ranvier, where action potentials are initiated. The AIS is crucial in information processing and transmission because the sum effect of integration of all synaptic inputs to a neuron culminates in one response: to produce an action potential or not. This output response is initiated at this heminode of Ranvier at the axon hillock, and the structure and ion channel composition of this heminode determine the threshold for action potential initiation and firing frequency. Studies described here illuminate mechanisms of plasticity at other nodes of Ranvier along a myelinated axon during learning. By initiating action potentials and determining the shape of the action potential, the AIS ultimately influences neurotransmitter release^29^. Also, the backpropagation of action potentials into the soma and proximal dendrites, which has a critical function in memory formation^30^, is determined by the length, precise location, and specialized proteins of the AIS^29^. The AIS exhibits activity-dependent plasticity that affects all of these aspects^29–31^. At the distal end of the axon, the terminal node of Ranvier, which depolarizes the nerve terminals to initiate neurotransmitter release, has a crucial function in synaptic transmission.

In addition to the mechanisms discussed thus far, oligodendrocytes can also alter action potential propagation by regulating extracellular ion concentration and releasing neurotransmitters. Depolarizing current injected into oligodendrocytes increases action potential conduction velocity in axons they myelinate in the CA1 region of hippocampus within seconds of stimulation. Theta burst stimulation of axons depolarizes oligodendrocytes via glutamate signaling and potassium channels^32^ (Fig. 1c, d).

After memory storage, retrieval of memories requires coupling together information stored in different neural circuits that are often separated by large distances in the brain. For example, episodic memory recall evokes a schema or a coherent cognitive representation of past events encompassing all the sensory, emotional, and situational information in the proper context and place where the past event was experienced and then replayed cognitively in the proper sequence. These various cognitive functions are processed and stored in distinct brain regions, which must be coordinately activated and combined in memory recall. It follows that if conduction velocities and axonal excitability are not optimal throughout the complex neural circuits that must be coordinately activated in memory recall, memory will be impaired. This leads to the conclusion that since conduction velocities are modifiable by alterations in myelin, then oligodendrocytes can orchestrate the coupling of transient neuronal assemblies for memory recall.

### Oligodendrocyte involvement in different types of learning

Learning can be broadly divided into associative and non-associative. The two are distinguished by a change in response to a repeated sensory stimulus (non-associative) versus responses that are altered by reinforcement or punishment (associative). The most elementary and ubiquitous forms of non-associative learning are habituation and sensitization, which are dampened or amplified responses (respectively) to a repeated stimulus. Sensitization and habituation are evident in animals with simple nervous systems and parallels are even seen in non-neuronal cellular responses; for example, cellular secretion or drug tolerance or sensitivity that develops from repeated stimulation.

In associative learning, responses are modified by pairing two stimuli; for example, a fear response is elicited by revisiting the location where a traumatic event occurred. Associative learning can be further categorized into declarative (explicit) and non-declarative (implicit) memory, which relate to how conscious awareness is involved. Learning is further categorized according to function, such as episodic memory to generate cinematic recall of a past experience; working memory to retain information temporarily to carry out a task; motor memory for developing physical skills, and others. These more complex forms of associative learning involve coordinating information stored in different parts of the nervous system responsible for the sensory, motor, and cognitive processes involved in the behavior.

Current evidence supports oligodendroglial involvement in many types of learning and memory, but most importantly, in those that have not received as much experimental attention. Historically, research on synaptic plasticity in memory has concerned rapid, simple reflex-type learning, such as operant conditioning, sensitization, etc. Many of these advances were made possible by isolated experimental systems, such as hippocampal brain slices, isolated cells, and invertebrates; notably *Aplysia*, studied in the context of simple forms of learning, such as gill-withdrawal reflex^33^ and hippocampal LTP and LTD in rodents. However, these experimental models preclude analysis of mechanisms involved in complex learning and recall at the network level where myelination has an important function in optimizing synchrony of spike time arrival and coupling neural oscillations across distant regions of the brain, for example, in learning complex motor skills, fear conditioning, and spatial memory among others. In addition, non-myelinating functions of oligodendroglia and plasticity of the nodes of Ranvier contribute to other types of learning by influencing intrinsic axonal excitability and synaptic function (Fig. 3).

#### Oligodendroglial involvement in non-associative learning

The rich body of research on invertebrates has identified diverse cellular mechanisms for habituation and sensitization, but oligodendroglia can alter intrinsic axonal excitability and synaptic function through alterations in nodes of Ranvier, the structure of myelin, effects of myelin on axon caliber, and by influencing extracellular ion concentration and neurotransmitters^32,34^.

To examine how changes in axon excitability, action potential waveform, and depolarization of nerve terminals to stimulate neurotransmitter release can be influenced by myelin in association with non-associative learning and short-term memory, consider that to enable saltatory conduction, voltage-gated sodium and potassium channels are situated along axons in tightly regulated spatial domains. Voltage-activated sodium channels are concentrated at the nodes of Ranvier and voltage-activated potassium channels of various types are expressed at the node of Ranvier and adjacent to it, to regulate excitability and the refractory period^35,36^. The action potential waveform, refractory period and action potential firing frequency, are dependent upon the proper structure and function of these ion channels, which is constrained by myelin flanking the node. When the myelin sheath retracts from the node of Ranvier through various experimental interventions, potassium channels are exposed and high frequency action potential conduction is impaired^37,38^.

A recent study reveals that detachment and reattachment of the paranodal loops of myelin flanking the nodes of Ranvier proceed under normal conditions, and that this can be regulated by astrocytes at the node of Ranvier^9^. In this study, the distribution of sodium channels in nodes of Ranvier broadened as the nodal gap lengthened from detachment of paranodal myelin, which is under control of exocytosis of thrombin protease inhibitors from perinodal astrocytes. The dispersion of sodium channels reduces the transmembrane sodium channel current density and together with increased membrane capacitance from the enlarged nodal gap width, slow action potential initiation and propagation rate (Fig. 3a). The detachment of the outer-most layer of myelin from the axon and resorption into the oligodendrocyte, also thins the myelin sheath to further slow conduction velocity. All of these effects can proceed in fully mature compact myelin and are reversible. Sleep deprivation^39^, hyperstimulation (noise exposure)^40^, spatial learning^41^ and disruption of GABAergic signaling^19^ also widen nodes of Ranvier, and while it is unclear whether astrocytes direct this response in each context, these studies highlight the widespread significance of activity-dependent nodal plasticity.

A contribution of myelin-independent functions of oligodendroglia in sensitization has been described^42^. In some brain regions, oligodendrocytes express glutamine synthetase, which is responsible for controlling levels of glutamine and glutamate, which are essential for cocaine-induced locomotor sensitization^42^. Conditional deletion of glutamine synthetase in OLs leads to a significant decrease in sensitization after cocaine injection (locomotor activity) without affecting myelination.

In addition to the influence of the myelin sheath on action potential propagation, axon diameter has a major influence on conduction velocity. Super-resolution imaging in hippocampus reveals activity-dependent plasticity of axon diameter that alters conduction velocity^43^. This can be a neuronal response, but oligodendrocytes also have a profound effect on axon caliber, as is most evident by striking changes in axon diameter at nodes of Ranvier. The effect of differences in axon caliber at nodes of Ranvier and differences in internodal length on action potential conduction velocity has been modeled mathematically^9^, with parallels found in auditory pathways conveying different frequencies of sound^44^.

#### Oligodendrocyte involvement in associative learning

Activity-dependent myelin plasticity is involved in many forms of associative learning. Some of the earliest studies of myelin in learning concerned motor learning, a procedural form of learning referring to the ability to gain new skills and to refine them by practice. These features make motor learning amenable to investigation using MRI brain imaging in humans by comparing differences in brain structure days or weeks after learning a skill. Although controversial at first, this body of research has established that there are structural changes in appropriate brain regions that accompany motor skill learning, reviewed elsewhere^45^. An unexpected finding was that changes were observed in white matter tracts after learning, not only in grey matter where synaptic plasticity occurs. This line of research, together with cell biological studies revealing that myelinating glia in the PNS^46^ and CNS^47^ can respond to action potential firing, stimulated research into the possibility that myelinating glia could participate in the cellular mechanisms of learning that involve transmission of information through white matter tracts^48^.

Animal studies show oligodendrocyte involvement in motor learning by using genetic manipulation to suppress the formation of new myelin in adult mice^49,50^. For example, Xiao et al.^50^, generated a conditional knockout of the transcription factor Myelin regulatory factor (*Myrf*) in OPCs and trained the mice to run on a complex wheel with random rungs missing. Deletion of *Myrf* arrests differentiation of OPCs, thereby blocking the production of new myelin-forming oligodendrocytes during adulthood without affecting preexisting oligodendrocytes or myelin^51,52^. Within 4 h of training, wildtype mice show improvement in their running performance, but *Myrf* knockout mice display poor performance in learning to run on the complex wheel. Motor learning is impaired in another myelin deficient mutant tested in a forelimb reaching task to obtain a food pellet reward^53^. Such studies show that motor skill learning involves the formation of new myelin.

Besides inducing myelination, motor learning influences oligodendroglia in other ways. For instance, motor learning remodels myelin structure by increasing the proportion of preexisting sheaths undergoing retraction and increasing the number of preexisting sheaths undergoing dynamic length changes^53^. OLs also influence motor learning through myelin-independent roles. OLs secrete and regulate brain-derived neurotrophic factor (BDNF)^54^. In another study, BDNF has been shown to promote motor learning^55^, but further research is needed to determine if BDNF derived from oligodendroglia affects learning and memory. Similarly, K^+^ channels in OLs control extracellular potassium clearance, and lack of potassium clearance by OLs enhances neuronal hyperactivity and induces seizures leading to poor motor performance^34^.

Several studies have reported OL contributions to fear memory, which also requires integrating information over distant brain regions. To investigate whether changes in myelin structure are associated with fear learning, Nguyen et al., generated hypomyelinated mice by knocking out the gene for tubulin polymerization promoting protein (*Tppp*) in OLs^56^. TPPP nucleates microtubules allowing transportation to and along the myelin sheath, leading to shorter and thinner myelin sheaths in KO animals. When studied in a cued fear conditioning task, in which aversive foot shocks are provided to mice in a novel environment to examine hippocampus, amygdala, and prefrontal cortex dependent associative learning, *Tppp* knockout mice exhibit impaired fear memory. The conditional deletion of *Myrf* from OPCs of adult mice, to prevent ongoing myelination, also affects fear conditioning^57^. Conversely, administering the drug clemastine, a compound that induces oligodendrogenesis and stimulates new myelin formation^58^, improves fear memory in wildtype mice^57^, further supporting the requirement of active myelination in fear memory.

The ability to orient in space is attributed not only to local circuitry within the hippocampus, but also requires communication between neurons in the hippocampus and cortical regions such as retrosplenial cortex^59^ and anterior cingulate cortex^60^, suggesting that spatial memory could involve myelinating glia to promote optimal synchrony of spike time arrival. Steadman et al.^22^, used the Morris water maze, in which mice are trained to locate a fixed platform, and after 3 days of training, spatial memory for the platform location is then tested. The results show that inhibiting the formation of mature oligodendrocytes in the adult mouse brain, by deleting *Myrf* in OPCs, does not weaken memory acquisition but impairs recall in the water maze. These findings are supported by an independent study that conditionally deleted the transcription factor *Olig2* in OPCs of young mice*. Olig2* is involved in regulating OPC differentiation and its deletion impairs spatial memory in water maze test^61^. Conversely, enhancing myelination by knocking out the muscarinic acetylcholine receptor type 1 (*Chrm1*), a negative regulator of oligodendrocyte differentiation^62^, improves spatial memory in older mice that have diminished myelin^61^. Improvement in the spatial memory test correlates with increased synapsin+ and vGlut1+ synaptic puncta in the CA1 hippocampal region. Thus, newly differentiated oligodendrocytes promote neurotransmission by influencing synaptogenesis in addition to forming new myelin. Spatial learning also remodels mature myelin structure by increasing node of Ranvier length and decreasing the periaxonal space^41^. Both alternations modify action potential conduction^63^.

#### Oligodendrocytes in working memory

Working memory provides a temporary storage of information necessary to carry out a cognitive function, and this depends heavily upon activity in frontoparietal brain regions. Electrophysiological studies in non-human primates, as well as EEG and fMRI studies in humans, indicate that working memory is dependent upon the persistent firing of neural networks that is sustained during the period the working memory is retained^64^. There is no compelling evidence that working memory is sustained by transient changes in synaptic strength, but obtaining this information is challenging (for review see ref. ^65^). Nguyen et al.^56^, report impaired working memory in *Tppp* KO mice that have myelin abnormalities, tested in a Y-maze. These mice have shorter segments of internodal myelin and thinner myelin, but the authors favor the view that impairments in information transmission negatively affect performance on the Y-maze test rather than involving new myelin sheath synthesis.

Studies in humans indicate that myelination contributes to working memory in many ways (for review see ref. ^66^). Through development^67^ performance of working memory increases in parallel with myelination of appropriate cortical brain regions^68^. Memory tests show that working memory peaks during adolescence and is maintained through early adulthood^69^ in parallel with development of these cortical regions, but in aging, cortical white matter begins to decrease in parallel with the normal weakening in working memory capacity in aging. Fractional anisotropy (an MRI measure related in part to myelin thickness), increases in the human brain in direct proportion to the hours of training in a working memory exercise, increasing linearly over the course of 2 months training^70^. Changes in fractional anisotropy are most prominent in intraparietal sulcus and anterior corpus callosum. In another study of working memory, similar changes are evident in the parietofrontal and parahippocampal regions^71^. This white matter plasticity could increase processing speed, firing frequency, bandwidth, and optimal oscillations required for working memory.

From such experiments, it is clear that oligodendroglia are involved in diverse types of learning, not only motor learning. In general, the effects of oligodendroglia in studies thus far are most evident in the types of learning that require transmission of information among distant brain regions through long projections and in complex learning tasks that require repetition and much longer time to develop as compared with simple reflexes or LTP.

### Oligodendrocytes participate in different phases of learning

**Fig. 4:**
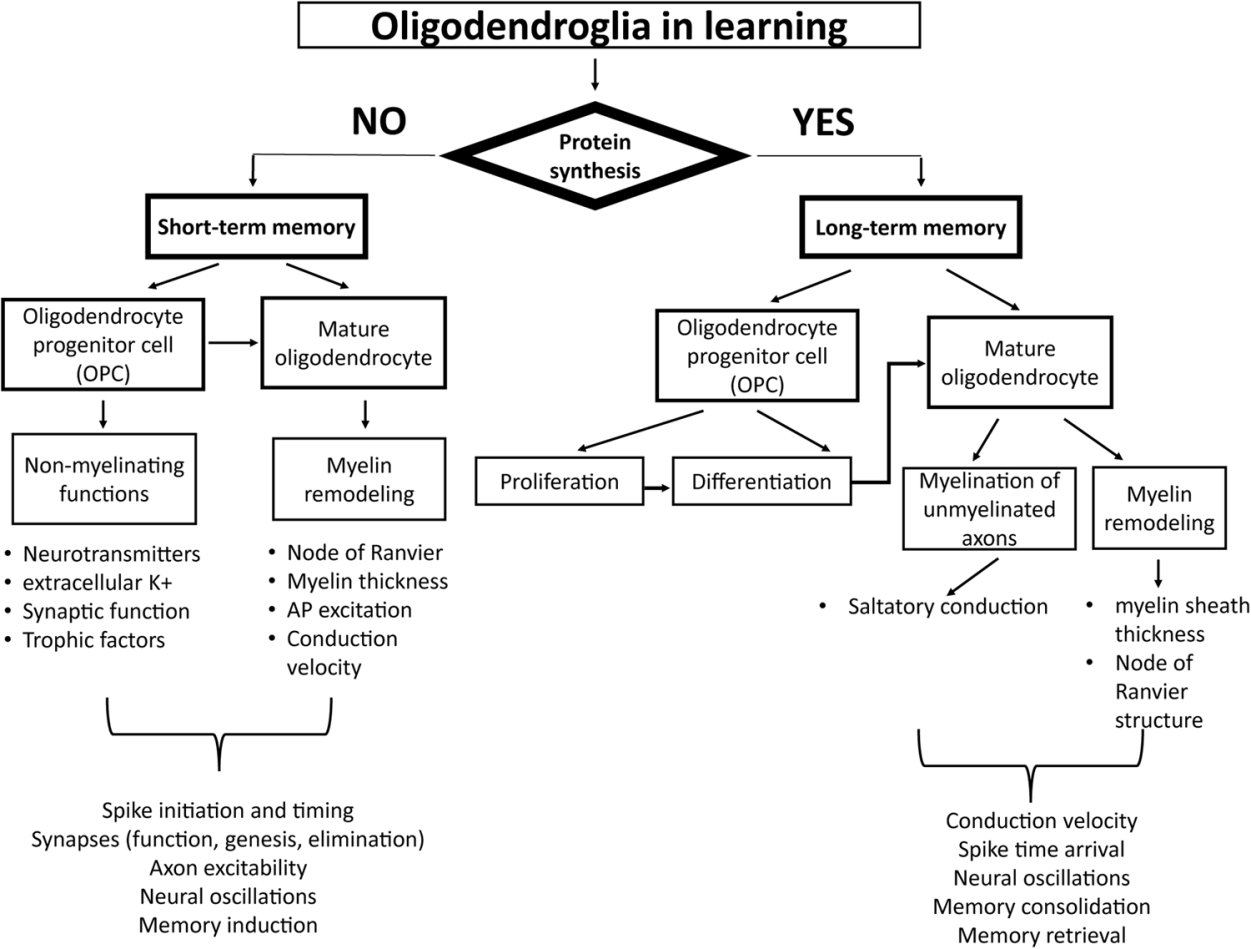
Oligodendrocyte involvement in short-term memory, long-term memory, and memory consolidation. The distinguishing factor between short- and long-term memory is the necessity of protein synthesis for long-term memory. Oligodendrocytes participate in short-term memory via changes in pre-existing myelin and through non-myelinating functions of oligodendroglial cells rather than formation of new myelin. Non-myelinating functions of OL and OPCs includes control of extracellular K^+^ concentration and regulating levels of neurotransmitters such as glutamate, essential for LTP and LTD. These changes affect axon excitability, and do not require protein synthesis. Activity-dependent initiation of myelination requires local protein synthesis (of myelin basic protein), indicating involvement of new myelin in long-term memory. Formation of new myelin and remodeling of mature myelin alter information processing and contributes to long-term memory. OPCs proliferate and differentiate into mature myelinating oligodendrocytes leading to new myelin sheath formation that contribute to remote memory consolidation by enhancing coupling among distance brain regions and within local circuits by optimizing spike time arrival and neural oscillations.

Several aspects of memory acquisition and recall are well established. Short-term memory is immediate but transient, whereas long-term memory requires repetition or an exceptionally novel or stressful event that evokes high levels of arousal, initiating so-called ‘flashbulb’ memory (for review see ref. ^33^). The cellular mechanisms for these two phases of memory differ, with local biochemical changes altering synaptic transmission in short-term memory, but long-term memory requires the synthesis of new proteins to support structural changes in synaptic connections^72^. Consolidation of short-term memory into long-term memory proceeds in the hours after a training session, and sleep is especially important in the process^73^. Finally, memory recall involves accessing information encoded by synaptic connectivity, but also assembling diverse memory stores in different brain regions and associating them with the current context. Several studies implicate oligodendroglia in long-term memory, but new research and theoretical considerations support oligodendroglial involvement in short-term memory as well (Fig. 4).

#### Oligodendroglia involvement in long-term memory

As mentioned above, oligodendrocytes have been implicated in long-term memory consolidation^22,57^. Both studies by Steadman et al.^22^, and Pan et al.^57^, used mice with a conditional deletion of *Myrf* gene in OPCs to prevent formation of new myelin, with the result that long-term memory deficits are evident in both spatial and fear memory. In fear conditioning experiments, both wildtype and *Myrf* knockout mice exhibit high levels of context-elicited freezing responses during recent retrieval sessions assessed 24 h after training. This indicates no effects of inhibiting oligodendrocyte maturation and *de nov*o myelination on short-term memory. However, freezing responses in *Myrf* knockout mice decline 30 days post-conditioning. This delayed memory deficit indicates that oligodendrocytes participate in consolidating remote fear memory^57^. Similarly, *Myrf* knockout mice search less selectively in the water maze when tested 28 days after training, but not during recent memory retrieval^22^. Electrophysiological recordings show that myelin formation in these mice increases the coordinated coupled oscillations between the hippocampus and medial prefrontal cortex to consolidate learning into long-term memory^22^.

Over the last 20 years, research has shown the significance of sleep on memory consolidation. During sleep, there is synchronized reactivation of neuronal ensembles in memory dependent brain regions such as hippocampus, neocortical areas, and striatal areas^74^. The neuronal connectivity in the hippocampus spreads to other regions, a process termed active systems consolidation (reviewed by^73^), in which short-term memory is consolidated into long-term memory. Such rhythmic coupling, essential for consolidation, would be achieved by ensuring precise spike-time arrival determined by appropriate conduction velocity established by myelin. Recent transcriptomic studies show that myelin-related genes are among the most common category of genes that change during sleep^39^. Upregulation of myelin-related genes essential for myelination and OPC proliferation occurs at night during REM sleep^75^. Likewise, chronic loss of sleep increases myelin thinning and widens nodes of Ranvier in mice^76^, further suggesting that myelin promotes synchrony of neural oscillations^22^ during memory consolidation in sleep.

#### Oligodendroglial involvement in short-term memory

Induction of short-term memory depends on the appropriate conduction velocity in axons, and thus myelin is important, for example, to support theta oscillations and synchronous spike time arrival for the induction of hippocampal LTP. Neural impulses can initiate myelination of unmyelinated axons rapidly, within 40 min, by vesicular release of glutamate from axons that activates NMDA and metabotropic glutamate receptors on oligodendrocyte process in close contact with axons^77^. This induces the formation of an axo-glial intercellular signaling complex that stimulates the local synthesis of myelin basic protein, without the need for gene transcription, to initiate formation of myelin preferentially on electrically active axons^78^. Studies in rodents and in zebrafish^79^ also show that myelination can be induced rapidly enough to participate in the induction and maintenance of short-term memory. However, the requirement for protein synthesis to initiate myelination excludes *de novo* myelination as a mechanism for short-term memory (Fig. 4). Present evidence indicates that the process of activity-dependent initiation of myelination is most consistent with involvement in consolidation of short-term memory into long-term memory.

There is evidence that non-myelinating functions of oligodendroglia can contribute to short-term memory (Fig. 4). Although the functional significance of the large population of OPCs in the adult brain remains unclear, non-myelinating functions of these progenitor cells could participate in short-term memory formation through release of trophic factors, phagocytosis of neurons and synapses, and modifications in synaptic transmission^80^. For example, OPCs express the transmembrane proteoglycan neuron-glia antigen 2 (NG2), and they are unique among glial cells in that they receive both glutamatergic^3^ and GABAergic^4^ synaptic input from neurons. Additionally, due to the presence of the large population of synapse-bearing OPCs in the adult brain^2^, OPCs can respond to neuronal activity in various ways, including by stimulating OPC differentiation and myelination, but also in other ways that are beginning to be explored. Intriguing studies by Sakry et al.^81^, show that OPCs not only respond to neuronal activity, they are also able to modulate it. Pyramidal neurons in the somatosensory cortex of NG2 knockout mice have reduced AMPA and NMDA receptor-mediated currents and greatly impaired LTP. This results from activity-dependent NG2 cleavage by the α-secretase ADAM10 in OPCs, which yields an ectodomain that is incorporated into the extracellular matrix. NG2-knockout mice have impaired LTP, and they exhibit altered behavior in tests of sensorimotor function, but not in contextual fear conditioning and water maze tests. The negative findings in the fear and spatial learning tests reported in this study warrant further investigation, considering heterogeneity of OPCs^82^ which can vary in different brain regions. For example, recent studies show that loss of NG2 protein in prefrontal cortex affects glutamatergic transmission^83^, and that NG2 modulates inhibitory neuronal activity through release of GABA^84^.

Other aspects of myelin plasticity have been described that could participate in short-term memory by altering axon excitability rather than synaptic transmission (Fig. 4). These involve mechanisms that change the structure of myelin and the nodes of Ranvier by cell biological mechanisms that do not depend on synthesis of new protein.

Disrupting the structure of mature myelin sheaths impairs short-term memory formation, but the results are open to different interpretations. A recent study shows that *Tppp* (tubulin polymerization promoting protein) knockout mice display short-term spatial and fear memory deficits 24-hr post-training in context-dependent fear conditioning and Y-maze tests^56^. These results are supported by another study that generated a conditional knockout of cyclin-dependent kinase 5 (Cdk5) in OLs^85^ resulting in thinner myelin sheaths and disrupted nodes of Ranvier. The Cdk5 KO mice display learning and memory deficits in behavioral tests such as the T-maze test for short-term spatial memory^85^. However, further validation is needed to determine whether myelin plasticity is a contributing mechanism to short-term memory or that hypomyelination or dysmyelination compromise optimal neural network function. Present evidence best supports the latter interpretation. In a looming fear assay, for example, the *Tppp* KO mice display decreased fear responses by spending less time hiding from a cast shadow^56^. This assay is a test of innate fear, which is not dependent upon learning and memory.

### Oligodendrocytes in memory impairments

Beyond the important advances in basic science by expanding research on learning and memory to understand the involvement of oligodendroglia and myelin plasticity, this new avenue of research has significant medical implications. Many psychological and neurological disorders are associated with learning and memory impairments throughout life, including developmental delay in children, cognitive impairment in aging, and in association with demyelinating diseases such as multiple sclerosis^86^. Because myelination proceeds actively through childhood and adolescence, and myelination is influenced by environmental experience, oligodendrocyte-based therapeutic approaches to address learning impairments in childhood would seem particularly fruitful. In addition to the ability of myelin regeneration in adulthood, the large number of OPCs in the adult brain provides a potential therapeutic target to improve memory in adulthood and in aging^87,88^.

One of the reasons that current treatments for memory disorders are not sufficiently effective may be that they are primarily based on augmenting synaptic transmission. A new approach shows that myelination can rescue cognitive decline associated with Alzheimer’s disease^89^, a neurological disorder characterized by myelin loss^90^ partially due to immature OPCs^88^. Note that not all types of OPCs differentiate into myelinating oligodendrocytes. Those that do are termed here as immature OPCs. The various subtypes of OPCs and their functions are matters of current investigation. Administration of clemastine, which promotes myelination, to Alzheimer’s mice improves performance in water maze and object recognition tests without altering their amyloid plaque content. These findings are supported by another study that utilized a different Alzheimer’s model and treatment to induce myelination^91^. Myelin therapies could complement other interventions, such as reducing beta-amyloid plaques to improve memory. While current studies show the benefit of using clemastine to improve cognition and memory generalization^55^, future studies are needed to further elucidate the cellular mechanisms of myelin renewal upon treatment and investigate its possible effect on amyloid plaques. A recent study indicates that clemastine induces myelin renewal by preventing senescence of OPCs^92^, coinciding with an increase in autophagy^93^.

## Summary and conclusions

High fidelity and stability of neural transmission are essential for neural network function, and this has always been recognized as the primary function of myelin, but experience-dependent optimization of transmission through complex neural networks is also necessary. Oligodendroglia influence information encoding, storage, and recall, by modifying action potential and synaptic transmission. Spike time arrival, intrinsic excitability of axons, and the frequency and phase coupling of neural oscillations are all subject to modifications by oligodendroglia. Through such modifications, activity-dependent myelination participates in long-term memory, memory consolidation, and recall in many types and phases of learning, including associative and non-associative learning. Current evidence best supports involvement of myelin plasticity in learning that involves repetitive trials and is most strongly associated with slower and longer-lasting effects on learning and memory, such as memory consolidation and recall. This is consistent with myelination being slow, in contrast to synaptic plasticity, which operates on millisecond time scales. In many respects, complex learning that requires prolonged practice and involves integration, storage, and recall of information among many brain regions, is the most interesting and relevant type of learning and memory for human function and dysfunction.

The extent to which oligodendroglia contribute to short-term memory is less clear at present because of a lack of experimental designs that have adequately tested this hypothesis. *De novo* myelination can be initiated rapidly within minutes of action potential firing, and alterations in nodal and paranodal structure can proceed rapidly, but the fundamental distinction between short-term and long-term memory is whether protein synthesis is necessary. Experimental evidence thus far indicates that *de novo* myelination does require local protein synthesis, but oligodendroglia can participate in short-term memory induction and maintenance by altering neurotransmitter and extracellular ion levels and neural oscillations.

### Critical appraisal and future directions

As a new field of research, which bridges traditionally separate scientific disciplines, a critical appraisal of present evidence and future directions of research on oligodendroglial involvement in learning and memory is appropriate. While new research is revealing the contributions of oligodendrocytes in learning and memory, new methods for investigating their involvement are needed. Plasticity of nodal structure, myelin thickness, and other structural and functional changes in myelin cannot always be obtained by assay of myelin protein or mRNA levels. Morphological, electrophysiological, and physiological analyses are required, but these approaches are considerably more difficult to perform in studying myelin plasticity than in synaptic plasticity because myelin structure and function span from the submicroscopic ultrastructure of myelin and function of nodes of Ranvier to the macroscopic expanse of myelin spanning the brain through white matter tracts. Specificity in morphological studies is particularly difficult, as electron microscopic analysis must be performed on the appropriate individual axons among thousands in white matter tracts and grey matter that are participating in the learning task.

Many studies of myelin in learning are observational, often with multiple types of learning tests performed together with other behavioral tests to identify a phenotype resulting from interrupting oligodendrocyte biology. While providing valuable information, the outcome from this approach can be difficult to interpret. In the future, experimental designs that test specific mechanistic hypotheses in appropriate brain regions responsible for the learning task are required. This will require better specificity of experimental interventions on specific aspects of oligodendroglia. For example, the use of clemastine fumarate or genetic approaches to induce remyelination lack regional and cellular specificity, which makes it harder to elucidate the effects of myelination in the brain regions associated with learning and memory. Similar limitations pertain to studies investigating contributions of oligodendrocytes by relying on the non-region-specific conditional deletion of genes such as *Myrf* to prevent OPC differentiation and myelination. A potential solution to this lack of specificity might be to develop optogenetic and chemogenetic tools to target OPCs and mature oligodendrocytes.

Physiologically relevant stimulation is an important consideration in advancing the field. Optogenetics, pharmacological treatments, transcranial magnetic stimulation (TMS), and direct depolarization to manipulate neural activity or oligodendroglial responses are quite artificial. These and other approaches provide important data, but the effects on myelin may be responses to pathological or abnormal conditions. Different animal models for investigating myelin involvement in learning and memory may be required. Conduction latencies are much greater in large brains, such as in primates, and in association with more complex cognitive processes that involve transmission and integration of information through multiple brain regions. Small organisms, such as mice and zebrafish, enable unsurpassed genetic manipulation and live imaging of myelination, but the conduction delays through the very small brains are minimal.

A confound in many experiments that implicate oligodendrocytes in learning by disrupting oligodendrocyte development or myelin integrity, is that impairments in optimal information transmission in neural networks could degrade performance in memory tests rather than myelin plasticity being a mechanism for learning. Experimental designs to separate these two aspects of myelin biology are needed. Distinguishing cause from effect and correlation from causation can be difficult in activity-dependent studies on myelin. Experimental perturbations that alter neurons, axons, or synapses, may induce secondary responses in oligodendroglia, rather than oligodendroglia having a primary function as a cellular mechanism of learning and memory.

There is an apparent bias in assuming that learning should increase myelination, but if achieving optimal spike time arrival is how myelin plasticity contributes to information processing and learning, then slowing conduction velocity in axons with latencies that are too short should be equally important. Thus, thinning the myelin sheath or other changes in nodes of Ranvier to slow conduction velocity would be expected. This greatly complicates analysis, because it requires axon-specific measures of myelination or nodal morphology rather than mean differences in sampled tissues, as learning would be expected to increase conduction velocity in some axons while reducing it in others to promote synchrony.

Our understanding of oligodendrocyte biology would benefit from better integration of experiments on myelin biology into the well-established field and nomenclature used in learning and memory research. Likewise, myelin plasticity contributes a new aspect to the learning and memory field by operating on large-scale, network-wide systems level and by proceeding over slower and more persistent periods. In the field of learning and memory, new nomenclature and possibly additional conceptual categorizations of types and phases of learning may be helpful in recognition of the new understanding of activity-dependent myelin plasticity.

Finally, the contribution of myelin plasticity in learning and memory is presumed to operate by optimizing the synchrony of spike time arrival in neural networks, yet there is no clear theory for how an oligodendrocyte could ‘know’ that optimal synchrony of spike time arrival at all relay points in a network has been achieved. In this sense, the field of activity-dependent myelination currently resembles the field of synaptic plasticity before Donald Hebb proposed his postulate in 1949 that guided decades of subsequent research and advances in synaptic plasticity in learning and memory.
